# Evaluation of the impact of breast milk expression in early postpartum period on breastfeeding duration: a prospective cohort study

**DOI:** 10.1186/s12884-015-0698-6

**Published:** 2015-10-20

**Authors:** Beiqi Jiang, Jing Hua, Yijing Wang, Yun Fu, Zhigang Zhuang, Liping Zhu

**Affiliations:** Department of Breast Disease and Breastfeeding Consulting, Shanghai First Maternity and Infant Hospital, Tongji University School of Medicine, 536 Changle Road, Shanghai, 200040 China; Shanghai Maternal and Child Health Center, 339 Luding Road, Shanghai, 200062 China

**Keywords:** Breast milk expression, Breast pumping, Breastfeeding duration, Survival analysis

## Abstract

**Background:**

Breast milk expression (breast pumping) has become prevalent as an important dimension of breastfeeding behavior. It is, however, not clear whether increasing breast milk expression contributes to extend the duration of breastfeeding. The objective of the present study was to evaluate the impact of breast milk expression in early postpartum period on breastfeeding duration amongst mothers of healthy term infants.

**Methods:**

A prospective cohort study had been conducted from March to June 2010. Mothers who gave birth to healthy, full-term and singleton babies were enrolled at discharge. These women were interviewed at 6 weeks postpartum about their breastfeeding behaviors. According to expressing patterns at 6 week postpartum, women were divided into three groups: direct breastfeeding (group 1), combining direct breastfeeding with expressing (group 2), exclusive expressing (group 3). The investigators followed up the women by telephone thereafter at a bimonthly basis and documented breastfeeding duration. Survival analysis was conducted to explore the association between expressing patterns at 6 weeks postpartum and breastfeeding duration. Associated factors of exclusive expressing at 6 weeks postpartum were characterized by logistic regression analysis.

**Results:**

Four hundred one eligible women were enrolled at discharge. Among the 389 women who attended the face-to-face interview at 6 weeks postpartum, 345 women continued breastfeeding. They were divided into 3 groups by their expressing patterns. According to survival analysis, women who exclusively expressed breast milk at 6 months postpartum (group 3) were 1.77 times as likely to stop breastfeeding as those who did not (group 1 and 2) (95 % confidence interval: 1.25–2.48; *P* <0.001). There is, however, no significant difference of breastfeeding duration between group 1 and group 2. Subgroup analysis showed that exclusive expressing women who were exclusively breastfeeding at 6 weeks postpartum had the shortest breastfeeding duration. Mother’s high education level, short maternity leave, breast milk expression in hospital and bottle-feeding in hospital were associated factors to exclusive expressing at 6 weeks postpartum.

**Conclusions:**

Exclusive expressing in the early postpartum period may not help women to achieve long-term breastfeeding duration, especially in women who were exclusively breastfeeding.

## Background

Breast milk expression (breast pumping) is widely practiced by mothers as an important dimension of breastfeeding behavior. Many experts and lactation consultants believe that breast milk expression is a helpful alternative for mothers to feed their babies breast milk when direct breastfeeding is not convenient or feasible, thereby allow mothers to continue breastfeeding and achieve their breastfeeding goals. Feeding a premature or unhealthy infant had been the main reason for using a pump to express breast milk in the past [1]. Breast pumps play an important role in promoting breastfeeding among working mothers [2]. A breast pump loan program involving 12,283 low-income women demonstrated an increase in the breastfeeding initiation from 1992 to 1996 in America [3]. Therefore, education of both breastfeeding and breast pump usage has been routinely provided by lactation consultants to new mothers for promoting breastfeeding [4]. Nowadays, expressing breast milk has become increasingly prevalent. A large proportion of mothers rely on breast pumps to feed their infants for health-related reason, early returning to work, and more importantly, convenience sake. Some mothers prefer exclusive milk expressing to direct feeding at breast, as a way of breast milk feeding immediately after giving birth [5–7]. As a result, the methods and patterns of breastfeeding are much more complicated than before. Breastfeeding behaviors reflect the thoughts and actions of a mother about breastfeeding, such as breastfeeding intention, confidence, persistence, and emotional reactions, which may in turn affect the decision to continue breastfeeding after returning to work and the overall breastfeeding duration. It is, however, not clear whether increasing breast pump usage really contributes to extend the duration of breastfeeding, or just complicates the life of the lactating women, as questioned by some health professionals [8–10]. The outcomes related to breast milk expression are not well understood so far, for the studies evaluating the impact of breast milk expression (breast pumping) on breastfeeding have generated inconsistent results. Win et al. reported that women who expressed breast milk were more likely to continue breastfeeding at 6 months than those who did not express [2]. Geraghty’s (2005) research, however, indicated that women who fed infants solely at the breast at early postpartum time points, were more likely to breastfeed for longer than women who combined direct breastfeeding with expressing or pumped only [11]. Interestingly, Geraghty’s another study (2012) suggested that breast milk expression by 4 weeks did not significantly influence duration of breast milk feeding [12].

Given these inconsistent evidences regarding breast milk expression and breastfeeding outcomes, we were interested to know the prevalence of breast milk expression in mothers who gave birth to healthy term infants in Shanghai, China. The aim of the present study was to evaluate the impact of breast milk expression in early postpartum period upon breastfeeding duration. Breastfeeding behaviors can vary over the early months postpartum. Six weeks postpartum is the time point when mothers establish their breastfeeding routine and still in maternity leave. Breastfeeding behavior during this period may have significant influence upon the decision of continuing breastfeeding after these mothers return to work. Therefore, breast milk expressing pattern at 6 weeks postpartum was selected as an exposure variable for the study.

Providing proper education program about breast pump usage has been another important subject. Chen PG.(2012) reported results from an analysis of the Infant Feeding Practices Survey II: there was a negative association between longer breastfeeding duration and receiving breast pump education from a physician/physician assistant; On the other hand, there were positive association between longer breastfeeding duration and receiving breast pump education from friends/relatives [4]. Our study may shed new light on the impact of breast milk expression upon breastfeeding outcomes, so that physicians can provide more effective advices to new mothers regarding breast pump usage, especially in their first stage of breastfeeding.

## Methods

### Study design

The study was conducted from March through June 2010, in Shanghai First Maternity and Infant Hospital, Tongji University School of Medicine. The hospital is a leading obstetrics and gynecology hospital in China, which provides maternity services covering all the districts in Shanghai. The obstetrics unit of the hospital has 400 beds in total, and delivers approximately 10,000 ~ 15,000 babies annually. This study was approved by the Institutional Review Board of Tongji University School of Medicine.

### Sample size

The primary outcome was mothers’ breastfeeding status and expressing pattern at 6 weeks postpartum, and the latter was an exposure variable in this study. In order to accurately evaluate the association between breastfeeding outcomes and expressing pattern at 6 weeks postpartum, a sample size of at least 425 women was estimated for this study. The estimation was based on the rate of breast milk expression of 62 % (pre-study survey of 800 women, prevalence of breast milk pumping at 6 weeks postpartum was 62 % [496/800], 95 % CI: 58.6–65.4 %), a power of 80 %, 2-tailed alpha of 0.05, and assuming 15 % of respondents lose visit.

Women were eligible to participate in the study if they met all of the following inclusion criteria: (1) gave birth to healthy, full-term, and singleton newborns in the hospital; (2) received prenatal breastfeeding education courses provided by the hospital; (3) ≥20 years of age; (4) had maternity leave >2 months.

Exclusion criteria were as follows: (1) contraindications to breastfeeding, such as HIV positive, active untreated tuberculosis, herpes simplex lesions on the breast, receiving antimetabolites or other chemotherapeutic agents; or (2) maternal illnesses that interfere with breastfeeding, such as Hepatitis B surface antigen-positive, Hepatitis C infection, diabetes or thyroid disease, or (3) mothers who had issues with alcohol use or smoke.

Many studies indicate that prenatal breastfeeding education is an associated factor for improved breastfeeding rate [13, 14]. To standardize the population in this dimension so as to reduce potential imbalance, the study required that all the enrolled women receive pre-delivery training provided by the hospital. There are researches show that alcohol intake and smoking have significant impact on the breastfeeding duration [15, 16]. In China, alcohol intake and smoking are not common among women and it is unusual for pregnant or breastfeeding women to consume alcohol or cigarette. To reduce the potential interference to the study outcome, we excluded the smokers and alcoholics as confounding factors which should be adjusted for in the study. Smokers were defined as those who ever smoke. Alcoholics were defined as those who consumed alcohol in pregnancy and would drink at least once a week during breastfeeding.

### Data collection

Within 24 h after giving birth, all women received routine postpartum care and breastfeeding initiation education from the lactation consultants in the ward, who documented information about enrollment. Based on these information, investigators determined the suitable cases and entered their admission number into SPSS on each recruitment day. Four hundred fifty women were sampled randomly from the 4500 eligible mothers with Random Processes (SPSS) and were invited to be enrolled into the study at discharge day (usually 2 or 3 days postpartum) by investigators. Informed consent form were signed off by the women if they agreed to participate in the study. These women were interviewed by investigators for their practices of breastfeeding initiation (information about pump use, bottle-feeding and formula supplementation during hospitalization). In this study, breastfeeding initiation is defined as any provision of the women’s own breast milk (expressed or directly from the breast) to the infant at least once between birth and hospital discharge. The baseline data regarding parity, baby’s gender, birth weight and type of delivery were taken from the medical records by investigators.

Detail face-to-face interview with the women were conducted when they visited the hospital for a postnatal examination and a routine check for newborns at 6 weeks postpartum. Data regarding primary outcomes of interest, breastfeeding status and expressing pattern at 6 weeks postpartum, were collected. Information on whether they had used a breast pump over the previous 1 week (during the period of 5–6 weeks postpartum) was interviewed and collected. Detail information of breast milk expression, such as the reasons for breast pump usage, expressing pattern, the positive and negative experience on expressing breast milk were documented.

The ultimate outcome was defined as the duration of breastfeeding, which is calculated as the total length of time a women produces breast milk (feeds her infant at breast or expresses breast milk). After the interview at 6 weeks postpartum, women were followed up over telephone at a bimonthly interval since the 2 months postpartum through breastfeeding cessation. Women were asked to recall their breastfeeding status and expression patterns in the past 1 week. The time point of the first time of using breast pump were documented if the women didn’t use pump before 6 weeks postpartum. Experiences on breast milk expression provided by women were recorded. The reason for breastfeeding cessation and the length of maternity leave were also documented.

### Variables

According to interagency group for action on breastfeeding (Labbok & Krasovec, 1990) [17], women’s breastfeeding behavior can be classified as exclusive breastfeeding (only breast milk, and no other types of milk is given to infants), partial breastfeeding (a combination of breast milk and formula milk is given to infants), and exclusive formula feeding (no breast milk is given to the infants). Here, the term “breastfeeding” is defined as infant feeding of breast milk, including milk expressed. It is important to further differentiate the expressing pattern, which was the exposure variable in this study. Women were classified into two groups based on their expression status at 6 weeks postpartum: direct breastfeeding group and expressing group. In this context, the term “ *breastfeeding*” is defined as the method of feeding a baby with milk directly from the women’s breast. Women who fed their infants solely at the breast and didn’t express breast milk by pumping were classified as the “*direct breastfeeding*” group (group 1). Those who used breast pumps to express milk were classified as the “*expressing*” group, which was further divided into “combining direct breastfeeding with expressing” (group 2) and “exclusive expressing” (group 3). In this paper, the term “*expressing*” (also known as “*pumping*”) is defined as using a pump to obtain breast milk, although manual expression is another way of breast milk expression. Manual expression was not included in this study because vast majority of the women were in possession of a breast pump and only a few women expressed milk by hand, as demonstrated by our study. If a woman expressed milk only by hand minimally or occasionally, she was classified into the “direct breastfeeding” group. For further survival analysis, women were divided into different subgroups: women who had a long maternity leave (>6 months) or a short maternity leave (≤6 months), women who were exclusively breastfeeding or those who were partial breastfeeding at 6 weeks postpartum.

Several maternal and infant factors as well as breastfeeding practices in hospital were assessed as covariates (predictors) in this analysis. These included mother’s age, education level, parity (primiparous, multiparous), infant gender, infant birth weight, and information of breastfeeding practice in hospital.

### Statistical analysis

Data were analyzed with SPSS 17.0. The percentage of mothers’ feeding method and rate of breast milk expression were calculated at various time points after giving birth. Kaplan-Meier and log-rank tests were adopted to assess the effect of some potential determinants on breastfeeding duration. Logistic analysis and Chi-square test were adopted to determine the associated factors that characterized mothers who chose to express milk exclusively at 6 weeks postpartum. Multiple logistic regression analysis was performed to identify a set of covariates with breastfeeding cessation in two models (breast cessation before 6 and 10 months after birth, respectively). Considering censored cases, multivariable Cox proportional hazards regression was employed to determine hazards ratios of covariates to breastfeeding duration. The covariates included in the multiple logistic analysis and multivariable Cox regression model were selected by an enter procedure, which enters all variables in the model in one single step without checking, therefore, to adjust for all the variables which were reported to be associated with breastfeeding duration by literatures. Kaplan-Meier survival curve and log-rank test were conducted for survival analysis and estimation of the difference between breastfeeding duration across the three groups of women who had different expressing patterns at 6 weeks postpartum and some subgroups. The significance level was set as 0.05.

## Results

Four hundred one randomly sampled women agreed to participate the study at discharge (response rate of 89.1 %, 401/450). The mean age (SD) of the 401 women was 29.2 (3.4) years. The mean (SD) birth weight of their infants was 3329.3 (490.1) g. 52.3 % (210/401) of the infants were born by cesarean section.

**Fig. 1:**
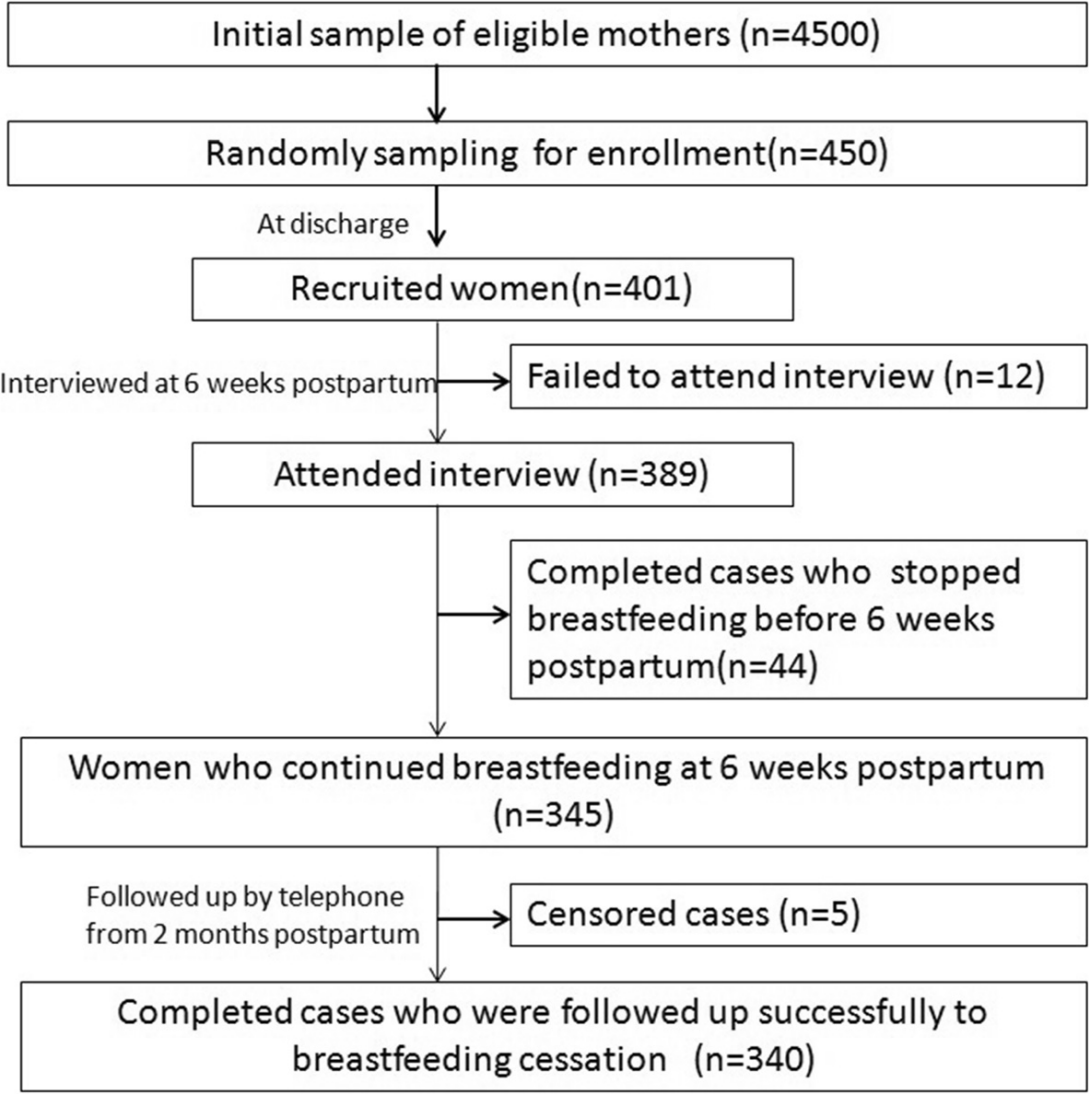
Recruitment details and study process

Three hundred eighty-nine women completed the interview at the postpartum check-up date of 6 weeks postpartum (response rate of 97 %, 389/401), and 12 women didn’t attend the interview, missing the key information for the study. During the follow-up period, 5 women lost visit while still keep breastfeeding (2 at 2 months postpartum, and 3 at 4 months postpartum), resulted in a sample of 384 women who had provided complete data of feeding practice and breastfeeding duration (Fig. 1). The study lasted for around 2 years until all the women ceased breastfeeding.

### Breastfeeding and breast milk expression during the first year after giving birth

**Fig. 2:**
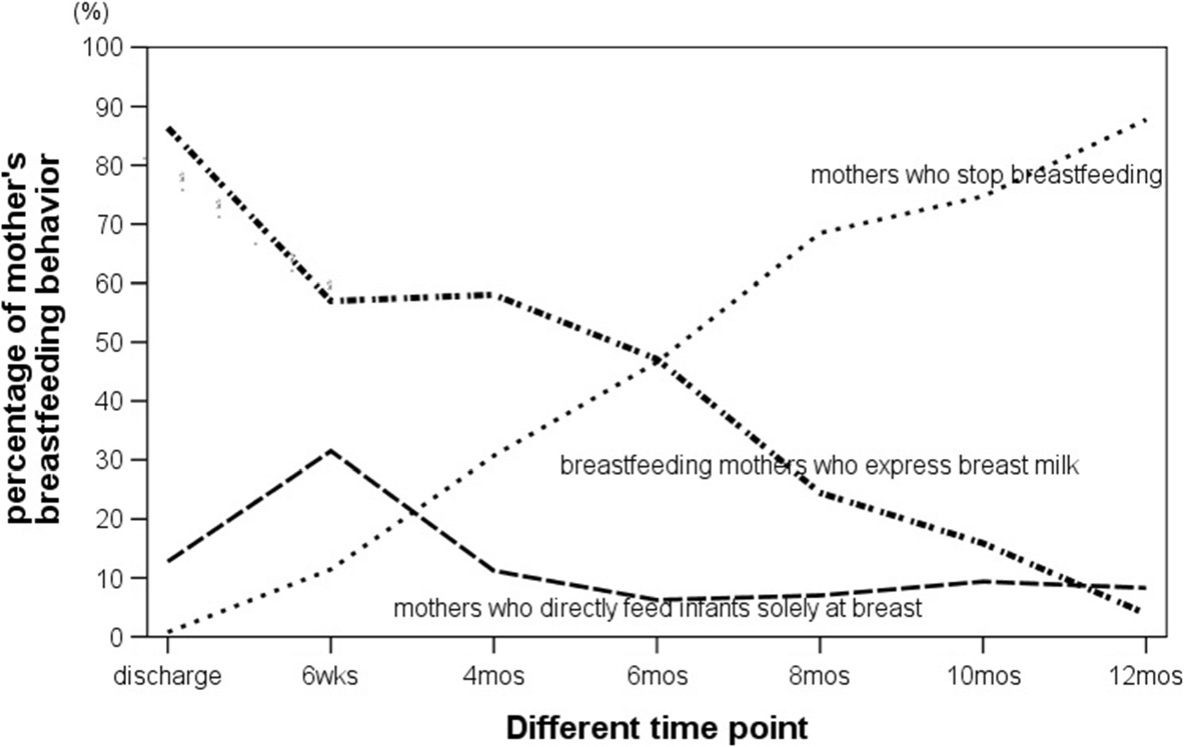
The percent change of mothers’ feeding method (expression status) at different time point in the first year after giving birth (*n* = 384). The percentage of expressing in women who breastfed was declined rapidly after 6 months postpartum, which suggested that breast pumping may not help in maintaining long-term breastfeeding

Table 1 shows the breastfeeding patterns and prevalence of breast milk expression during the first year after giving birth, based on the completed data of 384 mothers. Breastfeeding initiation was 99.2 %. Any breastfeeding and exclusive breastfeeding rates at 6 months were 51.8 and 6.8 %, respectively. The mean (SD) and median breastfeeding duration were 5.7 (3.3) and 6.1 months, respectively. Figure 2 shows the percentage change of mother’s feeding method (expression status) at different time point postpartum. The percentage of expressing in women who breastfed declined rapidly after 6 months postpartum, which suggested that breast milk pumping may not help in maintaining long-term breastfeeding.

**Table 1:** Patterns of breastfeeding and prevalence of breast milk expression during the first year after giving birth (*n* = 384)

	At discharge	To 6 weeks	To 4 months	To 6 months	To 8 months	To 10 months	To 12 months
Exclusive formula feeding	3 (0.8)	44 (11.5)	118 (30.7)	185 (48.2)	274 (71.3)	382 (85.4)	355 (92.4)
Any breastfeeding, *n* (%)	381 (99.2)	340 (88.5)	266 (69.3)	199 (51.8)	110 (28.7)	56 (14.6)	29 (7.6)
Exclusive breastfeeding, *n* (%)	69 (18.0)	132 (34.4)	76 (19.8)	26 (6.8)	0	0	0
Breast milk expression in breastfeeding mothers, *n* (%)	332 (87.1)	219 (64.4)	223 (83.8)	175 (87.9)	85 (77.3)	26 (46.4)	5 (17.2)

Demographic and clinical characteristics of the 389 women, including censor data were shown in Table 2. During hospitalization, 89.9 % (350/389) of them were in possession of a breast pump (45.7 % manual and 54.3 % electric or battery-operated). 85.9 % (334/389) of women expressed breast milk at least once in establishing breastfeeding. Common reasons for expressing milk in this period were postpartum engorgement (66.8 %), latch difficulties or baby’s refusal to suck (55.7 %), recommendation by others (35.9 %), and flat or inverted nipples (5.1 %).

**Table 2:** Mean breastfeeding duration in relation to maternal and infant characteristics (*n* = 389)

Variable	*N* (%)	Mean breastfeeding duration, months (95 % CI)	*P*
Maternal age (years) (*n* = 389)			0.41
20–25	48 (12.3)	5.8 (4.8–6.9)	
26–29	226 (58.1)	5.9 (5.5–6.3)	
30–35	93 (23.9)	5.6 (5.0–6.2)	
>35	22 (5.7)	4.3 (2.7–6.0)	
Baby gender (*n* = 389)			0.48
Male	206 (53.0)	5.6 (5.2–6.1)	
Female	183 (47.0)	5.9 (5.4–6.4)	
Birth weight (*n* = 389)			0.20
<2500 g	18 (4.6)	5.0 (3.6–6.3)	
2500–3999 g	348 (89.5)	5.8 (5.5–6.2)	
≥4000 g	23 (5.9)	5.0 (3.7–6.2)	
Primiparity (*n* = 389)			0.07
Yes	364 (93.6)	5.6 (5.3–5.9)	
No	25 (6.4)	7.4 (6.4–8.5)	
Delivery method (*n* = 389)			0.003
Vaginal	180 (46.3)	6.2 (5.7–6.7)	
Cesarean	209 (53.7)	5.4 (5.0–5.8)	
Education level (*n* = 389)			0.001
Graduate	23 (5.9)	3.9 (2.9–4.9)	
University/college	246 (63.2)	5.6 (5.2–6.0)	
Middle school	120 (30.8)	6.3 (5.7–6.0)	
Maternity leave (*n* = 389)			<0.001
>6 months	66 (17.0)	9.0(8.2–9.7)	
≤6 month	323 (83.0)	5.1(4.8–5.4)	
Bottle-feeding in hospital (*n* = 389)			0.03
Yes	350 (90.0)	5.6(5.3–5.9)	
No	39 (10.0)	7.1(6.1–8.2)	
Breast milk expression in hospital (*n* = 389)			0.47
Yes	334 (85.9)	5.7(5.3–6.0)	
No	55 (14.1)	6.1(5.2–7.0)	
Exclusive breastfeeding at 6 weeks postpartum (*n* = 345)			0.01
Yes	135 (39.1)	6.7 (6.2–7.3)	
No	210 (60.9)	6.1 (5.8–6.5)	
Expressing patterns at 6 weeks postpartum (*n* = 345)			<0.001
Direct breastfeeding (group 1)	123 (35.7)	6.7 (6.2–7.3)	
Combining direct breastfeeding with expressing (group 2)	144 (41.7)	6.7 (6.2–7.2)	
Exclusive expressing (group 3)	78 (22.6)	5.1 (4.6–5.6)	
Expressing patterns in exclusive breastfeeding mothers at 6 weeks postpartum (*n* = 135)			<0.001
Direct breastfeeding (group 1)	41 (30.3)	6.9 (5.8–7.9)	
Combining direct breastfeeding with expressing (group 2)	77 (57.0)	7.3 (6.6–8.1)	
Exclusive expressing (group 3)	17 (12.6)	3.8 (3.1–4.5)	
Expressing patterns in partial breastfeeding mothers at 6 weeks postpartum (*n* = 210)			0.02
Direct breastfeeding (group 1)	82 (39.0)	6.7 (6.1–7.3)	
Combining direct breastfeeding with expressing (group 2)	67 (31.9)	6.0 (5.4–6.7)	
Exclusive expressing (group 3)	61 (29.0)	5.5 (4.9–6.0)	

At 6 weeks postpartum, among 345 women who continued breastfeeding, 123 women fed infants solely at the breast (direct breastfeeding group, group 1). Two hundred twenty two (64.3 %) women used breast pump to express milk (expressing group). 144 women combined direct breastfeeding with expressing (group 2) for a variety of reasons, including need to empty breast due to oversupply of milk (58.3 %), to store extra milk (38.0 %), baby’s reluctance to suck at the breast (26.7 %), to increase milk supply (26.4 %), to allow the baby to be fed by someone other than the mother (17.4 %), painful nipples (21.5 %), mastitis (9.7 %), and flat nipples (3.5 %). 78 women expressed milk exclusively to feed infants via bottle (group 3), which corresponded to an exclusive milk expressing rate of 22.6 % (78/345). Exclusive expressing was more prevalent in women who were partial breastfeeding than in those who were exclusively breastfeeding (28.9 % vs 12.9 %, *p* <0.001). Baby’s refusal to suck (88.5 %) was the main reason for exclusive expressing. Only 23.1 % women chose expressing breast milk exclusively to feed infants because of flat or inverted nipples. Other reported reasons for exclusive expressing were as follows: allow baby to be fed by someone other than the mother (30.8 %), mastitis (21.8 %), and plan to return to work within coming 2 months (20.5 %).

**Fig. 3:**
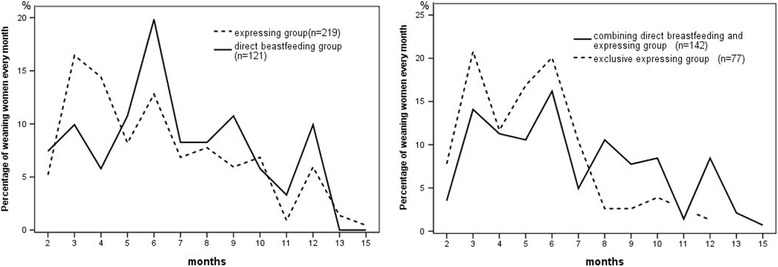
Weaning dynamics of women with different feeding method at 6 weeks postpartum (*n* = 340). Weaning dynamics was different in breastfeeding women who had different expressing pattern at 6 weeks postpartum. An earlier peak of breastfeeding cessation occurred in “expressing” group, compared in “direct breastfeeding” group. In “expressing” group, more exclusive expressing mothers stopped breastfeeding before 6 months than those who combined direct breastfeeding with expressing

Figure 3 shows that women with different breast milk expressing patterns at 6 weeks postpartum had different weaning dynamics beyond 6 week postpartum (group 1 versus group 2 + 3, group 2 versus group 3). Only 20.5 % (16/78) of exclusive expressing women continued breast milk feeding beyond 6 months after giving birth. The mean breastfeeding duration of exclusive expressing group was much shorter than that of women who combined direct breastfeeding with expressing (5.1 vs 6.7 months, *P* <0.001).

### Impact of expressing patterns at 6 weeks postpartum on breastfeeding outcomes

By logistic regression analysis , the factors associated with shorter breastfeeding duration were cesarean delivery, high education level (graduate), short maternity leave, bottle-feeding in hospital, breast milk expression in hospital, partial breastfeeding at 6 weeks postpartum and exclusive expressing at 6 weeks postpartum(Table 2 ).Table 3 showed the regression analysis of the expressing pattern as an exposure variable to breastfeeding outcomes, after adjusted for these associated factors and some other maternal and infant characteristics., Cox regression showed that women who expressed breast milk exclusively at 6 weeks postpartum (group 3) were 1.77 times as likely to stop breastfeeding as those who did not (group 1 + 2) (95 % confidence interval: 1.25–2.48, *P* <0.001). When comparing the two models of logistic regression, we find that exclusive expressing at 6 weeks postpartum was associated with increased likelihood of breastfeeding cessation before 6 month (adjusted odds risk 2.76, 95 % confidence interval 1.33–5.72, *P* = 0.01), but was not a significant risk factor to breastfeeding cessation before 10 months (*P* = 0.12).

**Table 3:** Associated risk factors to breastfeeding outcomes in women who breastfed more than 6 weeks

Variables	6 weeks ≤ breastfeeding <6 months (*n* = 340)^b^		6 weeks ≤ breastfeeding <10 months (*n* = 340)^b^		Breastfeeding duration (*n* = 345)	
	Adjusted^a^ OR (95 % CI)	*P*	Adjusted^a^ OR (95 % CI)	*P*	Adjusted^a^ RR (95 % CI)	*P*
Breast milk expressing pattern at 6 weeks		0.02		0.73		0.003
Exclusive expressing (group 3)	2.76 (1.33–5.72)	0.01	2.71 (0.78–9.37)	0.12	1.77 (1.25–2.48)	0.001
Combining direct breastfeeding with expressing (group 2)	1.38 (0.74–2.59)	0.31	0.99 (0.44–2.24)	0.99	1.08 (0.82–1.43)	0.58
Direct breastfeeding (group 1)	Reference		Reference		Reference	
Breast milk expression in hospital	0.80 (0.45–2.36)	0.80	0.96 (0.34–2.75)	0.94	0.85 (0.57–1.27)	0.44
Bottle-feeding in hospital	0.90 (0.38–2.13)	0.96	1.66 (0.55–5.04)	0.37	0.98 (0.63–1.51)	0.91
Maternity leave >6 months	0.6 (0.02–0.17)	<0.001	0.16 (0.08–0.31)	<0.001	0.38 (0.28–0.52)	<0.001
Exclusive breastfeeding at 6 weeks	0.89 (0.53–1.49)	0.65	1.69 (0.85–3.37)	0.13	1.09 (0.86–1.40)	0.48

^a^Multiple logistic regression and Cox regression (enter method) were adjusted by expressing patterns at 6 weeks postpartum, expressing in hospital, bottle-feeding in hospital, maternity leave, partial breastfeeding at 6 weeks postpartum, parity, delivery method, education level, mother’s age, baby gender and birth weight

^b^Among 340 women who breastfed more than 6 weeks, 199 mothers continued breastfeeding beyond 6 months postpartum, and 56 mothers continued breastfeeding beyond 10 months

**Fig. 4:**
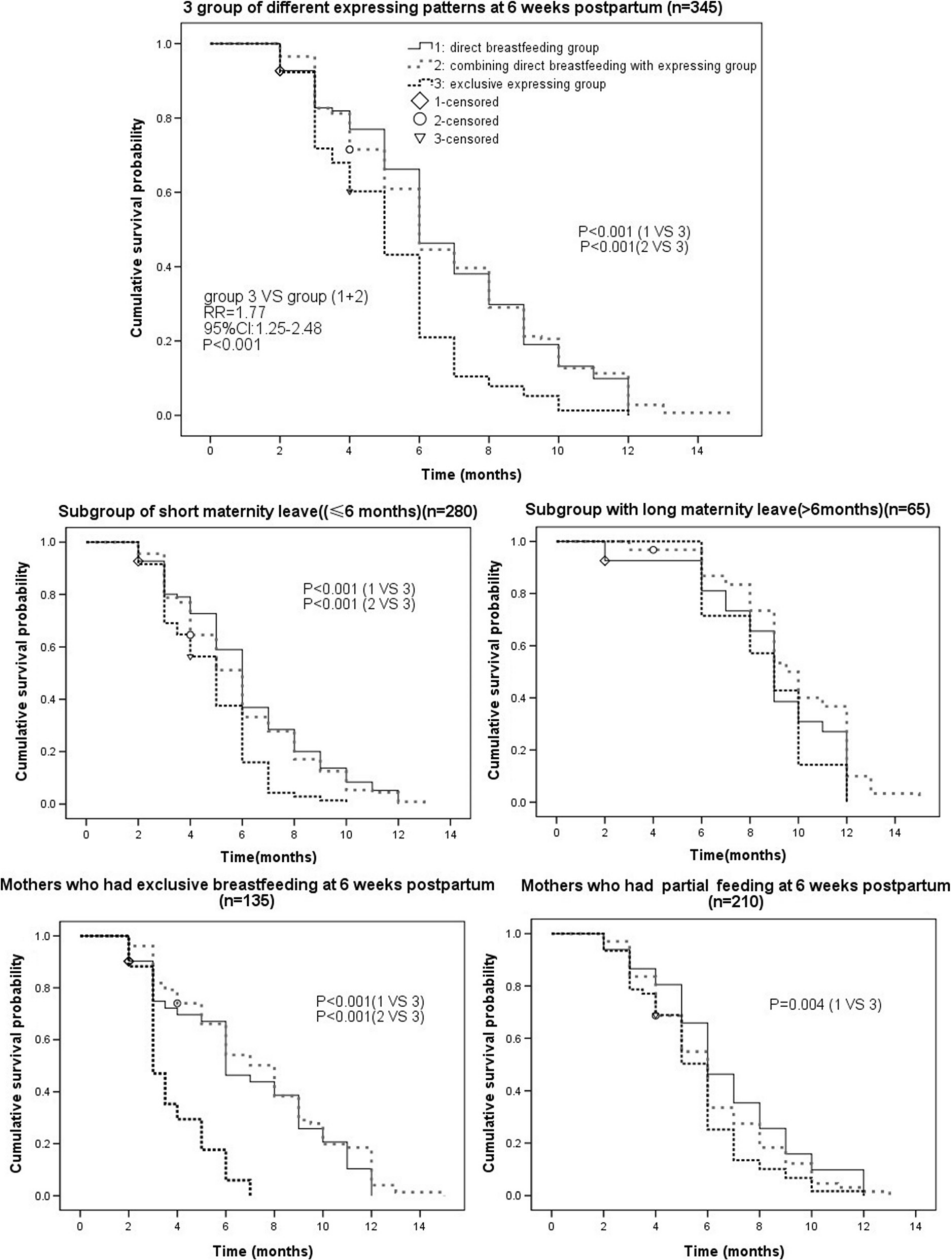
Kaplan-Meier survival plots showing the impact of expressing patterns on breastfeeding duration in the sample of 345 women and some subgroups. Survival curves of 3 groups were compared by a log-rank test

Although exclusive expressing at 6 weeks postpartum was associated with a shorter duration of breastfeeding, women who didn’t use a breast pump (group 1) and those who combined direct breastfeeding with expressing (group 2) had comparable breastfeeding duration by Kaplan-Merier survival analysis and log-rank test . In subgroup analysis (Fig. 4), exclusive expressing women who were exclusively breastfeeding at 6 weeks postpartum had the shortest breastfeeding duration. It is worth noting that no statistically significant difference in overall breastfeeding duration was detected across the 3 groups of different expression patterns in subgroup of those women who enjoyed long maternity leave (>6 months).

The descriptive characteristics of the women who expressed breast milk exclusively at 6 weeks postpartum versus those who did not were compared by logistic analysis (Table 4). High education level, short maternity leave (≤6 months), bottle-feeding and breast milk expression in hospital were shown to be associated with exclusive expressing at 6 weeks postpartum. A higher proportion of women who practiced partial breastfeeding were likely to choose exclusive expressing at 6 weeks postpartum.

**Table 4:** Odds ratios for factors associated with exclusive expressing at 6 weeks postpartum (*n* = 345)

Variables	Overall	Exclusive expressing		OR (95 % CI)	*P*
	*n *(%)	Did not (*n* = 267)	Did (*n* = 78)		
Maternal age (years)					
20–25	43(12.5)	36	7	0.27 (0.07–1.11)	0.07
26–30	206(59.7)	164	42	0.36 (0.11–1.19)	0.10
31–35	84(24.3)	60	24	0.56 (0.16–1.94)	0.36
>35	12(3.5)	7	5	Reference	
Delivery method					
Vaginal	159(46.1)	125	34	1.10 (0.67–1.81)	0.71
Cesarean	186(53.9)	142	44	Reference	
Baby gender					
Male	180(52.2)	138	42	0.92 (0.55–1.52)	0.74
Female	165(47.8)	129	36	Reference	
Birth weight					
<2500 g	16(4.6)	10	6	1.68 (0.40–7.08)	0.48
2500–3999 g	310(89.9)	243	67	0.28 (0.27–2.23)	0.63
≥4000 g	19(5.5)	14	5	Reference	
Education level					
Graduate	16(4.6)	7	9	4.92 (1.66–14.62)	0.01
University/college	218(63.2)	172	46	1.02 (0.58–1.80)	0.94
Middle school	111(32.2)	88	23	Reference	
Maternity leave					
>6 months	65(18.8)	58	7	0.36 (0.16–0.81)	0.01
≤6 month	280(81.2)	209	71	Reference	
Primiparity					
Yes	321(93.0)	252	69	Reference	0.08
No	24(7.0)	15	9	0.46 (0.19–1.08)	
Bottle feeding in hospital					
Yes	308(89.3)	231	77	Reference	0.01
No	37(10.7)	36	1	0.08(0.01–0.618)	0.01
Expressing milk in hospital					
Yes	297(86.1)	221	76	7.91 (1.88–33.37)	0.01
No	48(13.9)	46	2	Reference	
Exclusive breastfeeding at 6 weeks postpartum					
Yes	135(39.1)	118	17		<0.001^a^
No	210(60.9)	149	61		

^a^ *X* ^2^ test

## Discussion

Despite of the WHO recommendation of exclusive breastfeeding for the first 6 months after giving birth, most mothers fail to follow this goal in China. Shanghai is amongst the cities with the lowest breastfeeding rate [18]. In this study, although around 98 % mothers initiated breastfeeding, any and exclusive breastfeeding rates at 6 months were only 51.8 % and 6.8 % respectively, which were far below the current national target (50 % of exclusive breastfeeding rate for 0–6 month old babies in China from 2011 to 2020 [19]). A recent study reported a similar breastfeeding situation at a tertiary hospital in Northeast England: 94 % of mothers started any breastfeeding and 66 % initiated exclusive breastfeeding. By 26 weeks postpartum, 47 % mothers were still breastfeeding, but <1 % were exclusively breastfeeding [20]. These data suggest that there are underlying factors to be understood for maintaining breastfeeding after successful initiation. With increasing prevalence in breast pump usage in breastfeeding practice, breast milk expression is worth of notice as a modifiable factor which may influence breastfeeding outcome.

In this study, 64.3 % of women used breast pumps and approximately 22 % of women expressed breast milk exclusively to feed infant via bottle at 6 weeks postpartum. Our study indicates that exclusive expressing in early postpartum period was associated with shorter breastfeeding duration. There are some explanation for the negative impact of exclusive expressing on breastfeeding duration.

First of all, babies are much more effective at emptying milk from their mother’s breast than any pump can actually be, though there is no evidence or study to suggest that pumping lowers the milk supply. The baby’s bond with the mother can itself help to trigger the milk-ejection reflex (MER) when they are in direct contact [21]. Using a breast pump to express milk, a woman is essentially using physical manipulation to simulate breastfeeding, thereby “trigger” the breasts into “letting down” by way of MER. From our experience, at the first stage of milk supply building, new mothers usually have more difficulties in yielding sufficient milk to meet a baby’s demand by using a manual pump or exclusive expressing less than 10 sessions per 24 h, compared to direct feeding at breast.

Second, breastfeeding women who use a breast pump usually have a lot of anxiety or misconceptions over their milk supply and quality. Dykes considered that women expressed breast milk because of doubt about the adequacy of their milk supply [22]. In our study, there was a higher percentage of women choosing exclusive expressing in partial breastfeeding than in exclusive breastfeeding at 6 weeks postpartum. Many women who were partially breastfeeding complained that they were concerned about undersupply and felt frustrated at the time of expressing milk. Some women described their reason of breastfeeding cessation as their expressed breast milk looked so thin and watery in the bottle, that they worried the milk was not nutritious enough for the growth of the babies. Such misconceptions may lead to early formula supplement and breastfeeding cessation. Women who fed infants solely at breast, however, didn’t share this type of confusion or misconceptions. A recent paper of Flaherman (2014) also described some negative experiences of pumping among mothers with milk supply concern and concluded that pumping may present as barrier to successful breastfeeding [23].

Third, women express breast milk to feed their babies via bottle, or bottle-feeding, is another risk factor for breastfeeding duration. Some studies have shown that an infant fed from a bottle, regardless of the type of milk, is deprived of the benefits of self—regulation of intake associated with direct breastfeeding, which may increase the risk of subsequent childhood obesity [24, 25]. This indicates that bottle-fed babies may have greater demand for milk from a bottle than from mother’s breast, thereby aggravates the mother’s anxiety of milk supply insufficiency, and increases the possibility of earlier supplementation of complementary foods or formula. Moreover, direct breastfeeding helps to build a lasting psychosocial bond between the mother and her infant. Feeding babies from milk bottle instead of mothers’ breasts implies less skin to skin contact and may allow women who expressed breast milk exclusively more determined to stop breastfeeding when they return to work. This helps to explain why exclusive expressing, but not combining direct breastfeeding with expressing, shortens breastfeeding duration significantly.

Why women choose exclusive expressing as an alternative to direct feeding babies at the breast, though most of them have good nipple conditions and are in a position to take care of their healthy infants during the maternity leave? According to our study, the most common reason for exclusive expressing was baby’s refusal to suck (88.5 %), instead of flat/inverted nipples (23.1 %). Those women who bottle-fed babies during hospitalization were more likely to continue expressing milk in the following weeks. There is causal relationship between babies’ refusal to suck at breast and women’s breastfeeding practice during hospitalization. Bottle-feeding may result in “nipple confusion” [26], for it is a completely different feeding method regardless of attempts to make bottle-feeding more closely resemble breast-feeding. Non-nutritive and nutritive sucking occur throughout one breastfeed process. Infants need to suck nonnutritively at the breast for several minutes until the MER occurs, because little milk is available before MER [27]. With bottle-feeding, infants get milk flow immediately when a teat is inserted into the mouth. In other words, bottle-feeding makes the babies’ prefer the teat flow that produces the most milk with the least effort to the nipple flow [28]_._ Therefore, if a baby gets used to sucking milk from a bottle at the beginning, he/she may develop a reluctance thereafter to suck at the breast. As a result women are obliged to express breast milk to feed infants.

A high prevalence of in-hospital formula supplementation (82 %) leads to high rate of bottle-feeding (90 %) in our hospitals. Many studies found that in-hospital formula supplementation negatively affects breastfeeding duration and exclusivity [29, 30]. The Baby-Friendly Hospital Initiative therefore encourages eliminating formula use during the birth hospitalization for healthy breastfeeding infants [31]. However, the prevalence of in-hospital formula supplementation is still high, ranging from 23 to 78 % reported by many studies in developed countries [32–34]. Most infant formula was introduced for non-medical reason. Therefore, continuous efforts should be made to reduce unnecessary and non-medically indicated formula supplementation of healthy breastfeeding newborns to promote breastfeeding and keep a check on the use of milk bottles. When formula is medically needed, it should be given in a small cup, a syringe, a tube or a spoon, rather than in a bottle.

Our study showed that those women who had ever used breast pump during their hospitalization were more likely to continue expressing milk at later stage. Thus, new mothers should be advised to limit their breast pump usage during the establishment phase of breastfeeding so as to reduce the possibility of exclusive expressing. However, it is a common recommendation by many nurses and lactation consultants, though not evidence-based, that expressing both breasts after breastfeeding is helpful for relieving engorgement and increasing low milk supply during breastfeeding initiation. A study of Chapman et al. concluded that use of breast pump did not improve milk transfer during the first 72 h postpartum and may negatively affect breastfeeding duration among primiparous women who gave birth by cesarean section [35]. There are other studies showed that, during the initial postpartum period before the onset of copious milk production, milk volumes were small and hand expression might be even more effective and comfortable, than expression by breast pumps [36, 37]. Unfortunately, hand expression of milk has been an under-utilized skill in our hospital. Nurses and lactation consultants should acknowledge the benefits of hand expression, encourage and teach the skill of hand expression to new mothers for their breastfeeding initiation.

Survival analysis indicated that exclusive expressing women who were exclusively breastfeeding at 6 weeks postpartum had the shortest breastfeeding duration. Our follow-up records showed that these women described their experience in breast milk pumping as a hard, tiring and time-consuming task. Some women using manual pumps suffered from serious wrist joint pain. Women with an over-abundant milk supply suffered from full, engorged breasts, plugged ducts, and mastitis more often than other breastfeeding women. These difficulties and challenges compromised these women’s enthusiasm in exclusive expressing, and in turn, gave up breastfeeding. Flaherman suggests that, if a mother decides to express breast milk to feed her infant, clinicians should assess mother’s experience shortly after she initiates pumping, as further management and counseling may be necessary [23].

The length of maternity leave is also shown to be associated with breastfeeding initiation and duration significantly [38–41]. In our study, short maternity leave was a more significant risk factor than exclusive expressing in maintaining long-term breastfeeding. If women enjoyed a long maternity leave (>6 months), expressing pattern had no negative impact on breastfeeding duration. Many women decided to stop breastfeeding before returning to work, though they had used breast pump to express breast milk at home in early postpartum period. Majority of exclusive expressing women gave up breast milk feeding at the end of maternity leave because they had to face a number of challenges, such as long distances commuting between home and the work place, negative societal attitudes from the employer, lack of time and private space for expressing milk at working place. Even some women continued breastfeeding by expressing milk at work place, they complained of quick drop in milk supply. Working mothers need strong social support, including legislative measures, to enable them to continue breastfeeding. The findings indicate that short maternity leave represents a big hurdle for women in keeping breastfeeding for the recommended duration. Fortunately, maternity leave in China has been extended from 90 to 98 days (14 weeks) since April 2012 [42], which may help to prolong breastfeeding duration in the near future.

There are several limitations to the present study. First of all, selection bias. This study was restricted to women whose infants did not require neonatal intensive care, those who intended to breastfeed and agreed to participate in the study. Second, telephone follow-up every 2 months may be less effective in capturing information because of potential recall bias on accurate timing of breastfeeding cessation. Third, there are several methods of milk expression (hand versus pump) and numerous types of breast pumps (manual pumps versus electric pumps, simultaneous versus sequential technique, and double versus single, etc.). Our study did not include hand expression, because only a few women expressed milk by hand during breastfeeding. The impact of different types of breast pumps was not stratified in our study. Moreover, the patterns of breast milk expression were very complicated. The frequency and purpose of pumping vary from woman to woman. It would be an enormously challenging task to evaluate the influence of different expression cessation or auxiliary function of breast expression upon breastfeeding. Purpose of breast milk expression may reflect some psychosocial factors, such as breastfeeding intention, confidence, breastfeeding self-efficacy, body attitude, and motivation to breastfeeding, which are associated with breastfeeding outcomes. These factors were not taken into consideration in the present study. In addition, Labbok and Krasovec proposed the different levels of partial breastfeeding as high, medium and low in their earlier works [17]. These authors also recommended having token breastfeeding (breastfeeding with little or no nutritional impact) as a separate category. We didn’t distinguish these levels of partial breastfeeding in this study. As a result, the different level of formula use was not adjusted in the study.

Despite these limitations, our study shed new light on the impact of exclusive expressing during early postpartum period upon breastfeeding duration. Health professionals and lactation consultants should provide advices more effectively regarding breast milk expression to new mothers, so as to avoid barriers for successful breastfeeding. If women choose exclusive expressing to feed infants, proper education and counseling about breast pumping should also be provided to manage variety of problems in their exclusive expressing practice and help them achieve a longer breastfeeding duration.

## Conclusions

In conclusion, the rate of exclusive breastfeeding was only 6.77 % at 6 months postpartum in this study, far below the current national target of 50 % in China. Exclusive expressing (as opposed to breastfeeding only at breast and combining direct breastfeeding with expressing) in the early postpartum period was associated with a shorter duration of breastfeeding, especially in women who were exclusively breastfeeding. Health professionals and lactation consultants should provide more effective advice regarding the usage of breast pump during early postpartum period, so as to help new mothers to achieve a successful long-term breastfeeding.
